# Modelling the impact of human behavior using a two-layer Watts-Strogatz network for transmission and control of Mpox

**DOI:** 10.1186/s12879-024-09239-7

**Published:** 2024-03-26

**Authors:** Qiaojuan Jia, Ling Xue, Ran Sui, Junqi Huo

**Affiliations:** College of Mathematical Sciences, Harbin Engineering University, 145 Nantong Street, Harbin, Heilongjiang, 150001 China

**Keywords:** Mpox virus, Human behavior, Two-layer network, Reproduction number, Mitigation strategies

## Abstract

**Purpose:**

This study aims to evaluate the effectiveness of mitigation strategies and analyze the impact of human behavior on the transmission of Mpox. The results can provide guidance to public health authorities on comprehensive prevention and control for the new Mpox virus strain in the Democratic Republic of Congo as of December 2023.

**Methods:**

We develop a two-layer Watts-Strogatz network model. The basic reproduction number is calculated using the next-generation matrix approach. Markov chain Monte Carlo (MCMC) optimization algorithm is used to fit Mpox cases in Canada into the network model. Numerical simulations are used to assess the impact of mitigation strategies and human behavior on the final epidemic size.

**Results:**

Our results show that the contact transmission rate of low-risk groups and susceptible humans increases when the contact transmission rate of high-risk groups and susceptible humans is controlled as the Mpox epidemic spreads. The contact transmission rate of high-risk groups after May 18, 2022, is approximately 20% lower than that before May 18, 2022. Our findings indicate a positive correlation between the basic reproduction number and the level of heterogeneity in human contacts, with the basic reproduction number estimated at 2.3475 (95% CI: 0.0749–6.9084). Reducing the average number of sexual contacts to two per week effectively reduces the reproduction number to below one.

**Conclusion:**

We need to pay attention to the re-emergence of the epidemics caused by low-risk groups when an outbreak dominated by high-risk groups is under control. Numerical simulations show that reducing the average number of sexual contacts to two per week is effective in slowing down the rapid spread of the epidemic. Our findings offer guidance for the public health authorities of the Democratic Republic of Congo in developing effective mitigation strategies.

**Supplementary Information:**

The online version contains supplementary material available at 10.1186/s12879-024-09239-7.

## Introduction

The Mpox virus (MPXV) is an orthopoxvirus that causes Mpox, a disease with symptoms similar to smallpox. The incubation period of Mpox ranges from five to 21 days. The febrile stage of Mpox, also called the prodromal phase, usually lasts for one to three days with symptoms including fever, severe headache, swollen lymph nodes, myalgia, and asthenia. The febrile stage is followed by the rash stage, which lasts for two to four weeks. The proportion of patients who die due to Mpox reaches the maximum value of 11% [1]. Mpox can spread among humans through direct contacts with the infectious rash, respiratory secretions through physical contacts, touching the body fluids, and vertical transmission. It is also possible for humans to be infected with Mpox by being scratched or bitten by the infected animals or consuming infected animals [1]. The pattern of contacts between individuals is crucial in determining the spread among humans [1].

On May 6, 2022, a new phase of Mpox outbreak began when the first case, not associated with travel from Africa, was reported in the UK [2]. On June 6, 2022, the World Health Organization declared the Mpox outbreak a pandemic [1]. As of July 6, 2023, 88,122 cases and 148 deaths have been reported by 112 countries and territories, with 1,496 confirmed cases in Canada [3]. The Centers for Disease Control and Prevention (CDC) issued the Health Alert Network Health Advisory in December 2023 to notify clinicians and health departments about the sexually associated human-to-human transmission of Clade I MPXV in the Democratic Republic of the Congo (DRC) [4]. Therefore, we need to study the underlying mechanism of Mpox transmission in Canada to reduce the risk of Mpox transmission in non-endemic countries.

To gain deeper insights into the transmission dynamics and develop accurate mathematical models for Mpox, more and more different types of models are being explored. Based on the assumption that each individual has an equal probability of contacting others, compartmental SIR and SEIR models are used to model the transmission dynamics of Mpox between humans and animals. The results show the conditions for both local and global stability using the Lyapunov functions and center manifold theory [5–10]. In addition, compartmental models reveal that mitigation measures can curb the spread of Mpox, and the epidemic dies out when the basic reproduction number drops below one in humans and animals [11]. Moreover, the compartmental models that incorporate both quarantine and vaccination have been proposed to describe the interaction of human-to-human transmission and animal-to-human transmission of the MPXV [12]. Network models have explained the influence of the MSM and the number of sexual partners in the spread of Mpox [13]. The analysis of the network model shows that controlling the spread of misinformation can reduce the spread of Mpox [14]. Machine learning network models have been used for surveillance and rapid identification of suspected cases [15–18]. However, heterogeneity of human behavior is rarely captured in the earlier studies on Mpox. Additionally, gaps in the comprehensive evaluation of mitigation strategies have hindered the development of more effective mitigation strategies. Using epidemiological models to characterize human behavior is helpful for projecting effective mitigation strategies.

Studies have shown that the heterogeneity of sexual contacts among humans has an impact on the transmission of infectious diseases [19–23]. However, existing models rarely study the effect of heterogeneous human behavior on the transmission of Mpox [1, 10]. The spread of infectious diseases fundamentally depends on the pattern of human contact [20]. MPXV is spread through animal-to-animal, animal-to-human, and human-to-human transmission [1]. Susceptible humans are mainly infected through human-to-human transmission, with MSM accounting for over 83% [24, 25]. In addition, the survey about MSM shows that they preferred pet dogs and cats such that the risk of animal-to-human transmission is increased [26]. Therefore, we classify the population into high-risk groups (HR) and low-risk groups (LR). The heterogeneity of human behavior is also implied by the number of sexual contacts [27]. We assume that animals are homogeneously mixed. We place animals and humans into different layers of a network based on the transmission of Mpox to sort out the main factor that drives the spread of MPXV inside each layer and between two layers.

Network models are more intuitive and accurate in predicting disease transmission through heterogeneous host populations [28]. A short average path length reduces the probability that an epidemic dies out before reaching distant nodes of the network [29]. On the other hand, the presence of densely connected clusters within a network can also reduce the epidemic size [30]. The Watts-Strogatz (WS) network we selected contains the above two characteristics of high clustering coefficient and scalability, describing the transmission of Mpox in the real world [29, 31].

Considering both heterogeneous networks of human contacts and homogeneous networks of animal bites, we develop a two-layer WS network model. One layer of the network represents the spread between humans with heterogeneous contacts. The other layer represents transmission between homogeneous animals. There is a coupling between the two layers, indicating the transmission of Mpox from animals to humans.

We develop a two-layer Watts-Strogatz network model to simulate the spread of Mpox in Canada. This model considers animal-to-animal, animal-to-human, and human-to-human transmission. We derive the basic reproduction number, which is positively associated with the heterogeneity among humans. We utilize the Markov chain Monte Carlo (MCMC) optimization algorithm to fit Mpox cases in Canada into our network model. We obtain trends in the effective reproduction number between May 18, 2022, and August 18, 2022. Additionally, we assess mitigation strategies before and after August 18, 2022. Furthermore, our model can provide guidance for containing other zoonotic diseases such as rabies, HIV, Ebola, and salmonellosis [1].

The rest of this work is as follows. In Sect. 2, we introduce the source of infection, transmission route, compartments, and the conception and establishment of the two-layer Watts-Strogatz network model. In Sect. 3, we compute the basic reproduction number following the next generation matrix approach. We perform the microscopic simulation of the two-layer Watts-Strogatz network model and evaluate mitigation strategies in Sect. 4. We conclude the contribution, discuss the limitations, and propose future work in Sect. 5.

## Materials and methods

This section consists of three parts: purpose, data sources, and model formulation.

### Purpose

The objective of this study is twofold: firstly, to assess the efficacy of mitigation measures implemented to curb the spread of Mpox in Canada, and secondly, to formulate a comprehensive plan for prevention and treatment aimed at controlling human-to-human transmission of new Mpox strains in the Democratic Republic of Congo by December 2023.

### Data sources

The number of reported Mpox cases in Canada from May 18, 2022, to August 18, 2022, is obtained from the Our World in Data [32]. The parameters are obtained from the World Health Organization [1]. The population data is obtained from Statistics Canada [33].

### Model formulation

We develop a two-layer Watts-Strogatz network model to evaluate the transmission of Mpox in a heterogeneous population based on the following assumptions about the transmission characteristics of Mpox. Next, we introduce the source of infection and the routes of transmission.

Source of infection: MPXV mainly exists in African animals (African squirrels, tree squirrels, Gambian kangaroos, dormice, etc.) in nature. Primates, including monkeys, chimpanzees, humans, etc. can be infected by contacting animals that are infected with MPXV. Animals and humans that are infected with MPXV are the sources of infection [14].

Transmission routes:

1. Animal-to-animal transmission. Susceptible animals are infected mainly by the contact with respiratory secretions. Susceptible animals can also be infected through pathological exudates and the blood of infected animals though biting [1, 34, 35].
2. Animal-to-human transmission. Human infection is mainly caused by contacting respiratory secretions, pathological exudates, blood, and other body fluids of infected animals, or by being bitten and scratched by infected animals, and also by consuming infected animals [1, 34, 36]. In 2003, the source of the Mpox outbreak in the U.S.A is traced to native prairie dogs with wild animals imported from Africa [36].
3. Human-to-human transmission. Human-to-human transmission of Mpox is caused by contacting humans infected with MPXV, including contacts with respiratory secretions from those infected individuals, skin lesions or genitals, prolonged face-to-face contact, along with their bedding and clothing [1, 37–39]. Human-to-human transmission of MPXV is common in Canada, suggesting that close contact is the main way of transmission [25]. Another feature is that a large number of cases diagnosed with Mpox are male, and a considerable part of cases are MSM, especially in Canada [40].
4. Human-to-animal transmission. The transmission of MPXV from humans to animals has not been confirmed [1]. The majority of contacts between animals and humans occur when animals are consumed by humans [5]. Therefore, our model does not consider the transmission of MPXV from humans to animals.

### Two-layer network model

A two-layer network-based model incorporating the characteristics of MPXV transmission is developed to assess the spread of MPXV in heterogeneous humans and animals.

The first network layer, $L1$ represents the transmission relationship among $N$ agents (humans). The agents on this layer are crowded and active. According to different probabilities of transmitting MPXV through contacts, the population can be divided into high-risk groups (HR) and low-risk groups (LR). Links between nodes on $L1$ represent sexual contacts. Node $i$ in HR is linked to node $j$ in HR with probability $c_{hh}$, and is linked to node $k$ in LR with probability $c_{lh}$, and is absorbed into HR. A link can transmit MPXV when a susceptible node and an infected node have effective contacts.

The second network layer, $L2$ represents the transmission among $M$ agents (animals). The agents on $L2$ of network include wild animals and pets. After the initial case appears, the links among agents are connected by the contacts with the infected agents.

**Fig.1:**
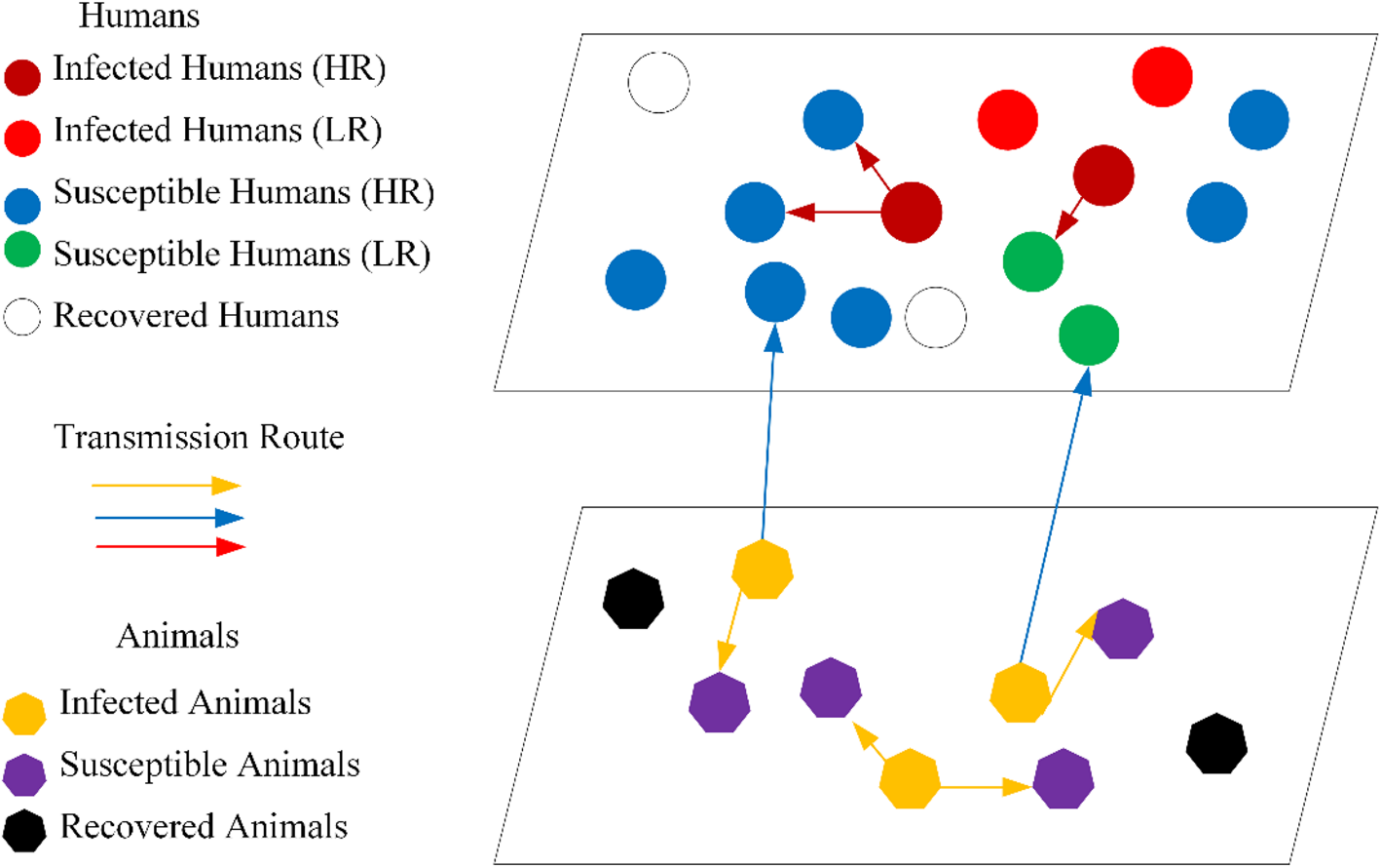
Schematic diagram of the network model. At any time $t$, nodes $i$ and $j$ are linked in three ways: nodes $i$ and $j$ are both in layer $L1$ at the same time, both in layer $L2$ at the same time, or one in layer $L1$ and one in layer $L2$. MPXV can be transmitted via the link if the infected individual in layer $L2$ has effective contacts with the susceptible individual in layer $L1$

In addition, there is interaction between the two layers of the network. The link between $L1$ and $L2$ can transmit MPXV only when the infected nodes in $L2$ has effective contacts with a susceptible node in $L1$. The schematic diagram of the network model is shown in Fig. 1.

Animals can be divided into the following four compartments: susceptible animals $\left( {S^{a} } \right)$, exposed animals $\left( {E^{a} } \right)$, infected animals $\left( {I^{a} } \right)$, and recovered animals $\left( {R^{a} } \right)$. The total number of animals keeps constant, denoted by $N^{a}$, which satisfies

$$N^{a} = S^{a} + E^{a} + I^{a} + R^{a} .$$

Humans are infected in a similar way as animals. Besides, humans have a fever period called the prodromal stage lasting for one to three days before the rash stage, and the symptoms include fever, severe headache, lymphadenopathy, and so on [1]. Therefore, humans can be divided into the following compartments: $S_{k}^{j}$ (susceptible), $E_{k}^{j}$ (exposed), $P_{k}^{j}$ (prodromal), $I_{k}^{j}$ (infected), and $R_{k}^{j}$ (recovered), where $j = h1,h2$ represent HR and LR, respectively.

For this network model, the degree of a node refers to the number of links connected to the node. The average degree of the network is given by

$$\left\langle k \right\rangle = \sum\limits_{k = 1}^{n} {kp\left( k \right)} ,\quad \;k = 1, \cdots ,n.$$

The animal-to-human transmission network is consisted of HR (of size $N^{h1}$), LR (of size $N^{h2}$) groups and animals are shown in Fig. 1. From the viewpoint of node degree, the network can also be divided into $n$ groups. $n$ represents the maximum number of sexual contacts per week. We have used a value of $n = 8$ in our numerical simulations [27]. Each group is a set of nodes with the same degree $k = 1, \cdots ,n$ of size $N_{k}^{h1}$ and $N_{k}^{h2}$, satisfying

$$N^{h} = N^{h1} + N^{h2} = \sum\limits_{k = 1}^{n} {N_{k}^{h1} } + \sum\limits_{k = 1}^{n} {N_{k}^{h2} } = \sum\limits_{k = 1}^{n} {\left( {S_{k}^{h1} + E_{k}^{h1} + P_{k}^{h1} + I_{k}^{h1} + R_{k}^{h1} } \right)} + \sum\limits_{k = 1}^{n} {\left( {S_{k}^{h2} + E_{k}^{h2} + P_{k}^{h2} + I_{k}^{h2} + R_{k}^{h2} } \right)} .$$

### SEPIR-SEIR epidemics on two-layer networks

The connections of individuals are formulated by the WS network. Animals are homogeneously mixed. The model we developed describes the spread of Mpox in the above two-layer network.

**Fig. 2:**
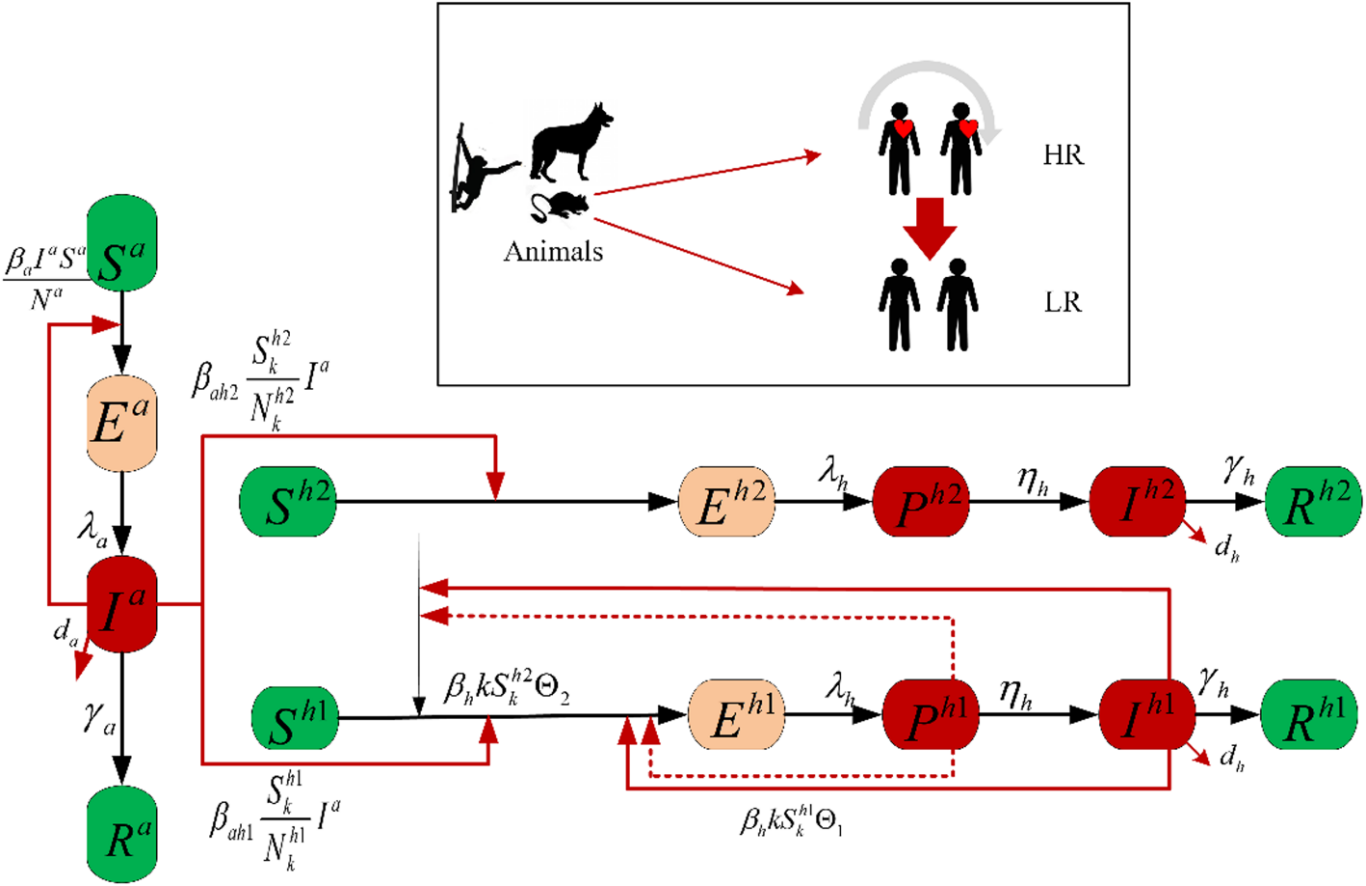
Schematic diagram of the Mpox transmission in both humans and animals [1, 24, 25]

Based on the above model settings, the susceptible humans in HR and LR are exposed to the prodromal stage $P$ and the infected stage $I$ of HR with probabilities, $\Theta_{1}$ and $\Theta_{2}$, respectively. The contact rate among HR is $\beta_{h}$. After the average incubation period ${1 \mathord{\left/ {\vphantom {1 {\lambda_{h} }}} \right. \kern-0pt} {\lambda_{h} }}$ days, exposed humans have a fever (prodromal). After the average incubation period ${1 \mathord{\left/ {\vphantom {1 {\lambda_{a} }}} \right. \kern-0pt} {\lambda_{a} }}$ days, exposed animals infected with Mpox. The average fever period of infected humans is ${1 \mathord{\left/ {\vphantom {1 {\eta_{h} }}} \right. \kern-0pt} {\eta_{h} }}$. Susceptible humans (HR and LR) and animals are infected by infected animals at the rates of $\beta_{ah1} ,\beta_{ah2}$, and $\beta_{a}$, respectively. A few severely infected humans and animals die at the rates, $d_{h}$ and $d_{a}$, respectively, while most recover at the rates of $\gamma_{h}$ and $\gamma_{a}$. The model following the schematic diagram in Fig. 2 is

$$\left\{ {\begin{array}{*{20}l} {\dot{S}_{k}^{h1} = - \beta_{h} kS_{k}^{h1} \Theta_{1} - \beta_{ah1} \frac{{S_{k}^{h1} }}{{N^{h} }}I^{a} ,} \hfill \\ {\dot{E}_{k}^{h1} = \beta_{h} kS_{k}^{h1} \Theta_{1} { + }\beta_{h} kS_{k}^{h2} \Theta_{2} { + }\beta_{ah1} \frac{{S_{k}^{h1} }}{{N^{h} }}I^{a} - \lambda_{h} E_{k}^{h1} ,} \hfill \\ \begin{gathered} \dot{P}_{k}^{h1} = \lambda_{h} E_{k}^{h1} - \eta_{h} P_{k}^{h1} , \hfill \\ \dot{I}_{k}^{h1} = \eta_{h} P_{k}^{h1} - \left( {\gamma_{h} + d_{h} } \right)I_{k}^{h1} , \hfill \\ \end{gathered} \hfill \\ \begin{gathered} \dot{R}_{k}^{h1} = \gamma_{h} I_{k}^{h1} . \hfill \\ \begin{array}{*{20}l} {\dot{S}_{k}^{h2} = - \beta_{h} kS_{k}^{h2} \Theta_{2} - \beta_{ah2} \frac{{S_{k}^{h2} }}{{N^{h} }}I^{a} ,} \hfill \\ {\dot{E}_{k}^{h2} = \beta_{ah2} \frac{{S_{k}^{h2} }}{{N^{h} }}I^{a} - \lambda_{h} E_{k}^{h2} ,\quad } \hfill \\ \begin{gathered} \dot{P}_{k}^{h2} = \lambda_{h} E_{k}^{h2} - \eta_{h} P_{k}^{h2} , \hfill \\ \dot{I}_{k}^{h2} = \eta_{h} P_{k}^{h2} - \left( {\gamma_{h} + d_{h} } \right)I_{k}^{h2} , \hfill \\ \end{gathered} \hfill \\ \begin{gathered} \dot{R}_{k}^{h2} = \gamma_{h} I_{k}^{h2} , \hfill \\ \begin{array}{*{20}l} {\dot{S}^{a} = - \beta_{a} \frac{{S^{a} }}{{N^{a} }}I^{a} } \hfill \\ {\dot{E}^{a} = \beta_{a} \frac{{S^{a} }}{{N^{a} }}I^{a} - \lambda_{a} E^{a} ,} \hfill \\ {\dot{I}^{a} = \lambda_{a} E^{a} - \left( {\gamma_{a} + d_{a} } \right)I^{a} ,} \hfill \\ {\dot{R}^{a} = \gamma_{a} I^{a} .} \hfill \\ \end{array} \hfill \\ \end{gathered} \hfill \\ \end{array} \hfill \\ \end{gathered} \hfill \\ \end{array} } \right.\quad \left( 1 \right)$$

Here,

$$\Theta_{1} = \frac{{c_{hh} \sum\limits_{k = 1}^{n} {k\left( {\xi_{1} P_{k}^{h1} + I_{k}^{h1} } \right)} }}{{\sum\limits_{k = 1}^{n} {kN_{k}^{h1} } }},\Theta_{2} = \frac{{c_{lh} \sum\limits_{k = 1}^{n} {k\left( {\xi_{1} P_{k}^{h1} + I_{k}^{h1} } \right)} }}{{\sum\limits_{k = 1}^{n} {kN_{k}^{h1} } }}.\quad \left( 2 \right)$$

The quantity $\Theta_{1} \left( {\Theta_{2} } \right)$ represents the probability that any given contact of high-risk (low-risk) individuals is linked to an infected high-risk individual.

In summary, our model is based on the three aspects below, which are verified in the numerical simulation section.

1. Sexual contact networks are neither regular networks nor random networks. The WS model is proposed by Watts and Strogatz [29]. The WS network model captures the features of short path lengths, high clustering coefficient, robustness, efficient information dissemination, flexibility and adaptability, and scalability, which are widely observed in real-world networks. Our model uses the WS networks to study the effect of human behavior and mitigation strategies on the transmission of Mpox.

2. We propose a novel individual-based network model that interconnects homogeneous animal vectors with heterogeneous human hosts. Moreover, our model can capture the effect of heterogeneous contacts of humans on MPXV transmission.

3. For human-to-human transmission, the probability of transmission among LR and from LR to HR is very small [24, 25]. Thus, we ignore the transmission paths from LR to HR and that among LR. We mainly focus on the transmission of MPXV among HR and that from HR to LR. We assume that LR transfers to HR after being infected.

### The parameter estimation

The parameter $\beta_{a}$ is the maximum value within the ranges given by literature [7]. Parameters $\lambda_{h} ,\lambda_{a} ,\eta_{h} ,\gamma_{h} ,\gamma_{a} ,d_{h}$, and $d_{a}$ can be inferred from data published by WHO in Table 1 [1]. $\Theta_{1}$ and $\Theta_{2}$ are calculated by substituting the values of other parameters into the formula (2).

**Table 1:** Values and definitions of parameters

Parameter	Definition	Value	Unit	Source
$\beta_{a}$	Transmission rate from infected	0.00125	Week^−1^	[7]
	animals to susceptible animals			
$\lambda_{h}$	Incubation period of humans with	1/2	Week^−1^	[1]
	a mean time of ${1 \mathord{\left/ {\vphantom {1 {\lambda_{h} }}} \right. \kern-0pt} {\lambda_{h} }}$ days			
$\lambda_{a}$	Incubation period of animals with	1/2	Week^−1^	[1]
	a mean time of ${1 \mathord{\left/ {\vphantom {1 {\lambda_{a} }}} \right. \kern-0pt} {\lambda_{a} }}$ days			
$\eta_{h}$	The length of the period between	7/3	Week^−1^	[1]
	prodromal phase and rash stage			
$\gamma_{h}$	Recovery rate of humans	1/3	Week^−1^	[1]
$\gamma_{a}$	Recovery rate of animals	1/3	Week^−1^	[1]
$d_{h}$	Mortality rate of humans	0.11	Week^−1^	[1]
$d_{a}$	Mortality rate of animals	0.11	Week^−1^	[1]

We use interpolating cubic splines in curve fitting. Interpolating cubic splines are widely used to fit a smooth continuous function through discrete data because they use low-order polynomials and have second-order parametric continuity, which yields a desirable smoothness constraint and monotonicity [41]. The 14 weeks are divided into four phases to determine the values of $\beta_{h} ,c_{hh}$, $c_{lh}$ and using a cubic function for each phase: Phase 1 is from May 18 to June 9, 2022, Phase 2 is from June 10 to June 30, 2022, Phase 3 is from July 1 to July 21, 2022, and Phase 4 is from July 22 to August 18, 2022. We parameterize the model with reported data on Mpox cases in Canada and assess the impact of mitigation strategies and contact heterogeneity on the epidemic in Canada.

Both the mean and variance of Poisson degree distribution,

$$P\left( k \right) = \frac{{\left\langle k \right\rangle^{k} e^{ - \left\langle k \right\rangle } }}{k!}$$

are equal to $\left\langle k \right\rangle$ [42]. Some humans have an awareness of protection as Mpox cases increase [43].

We simulate the spread of Mpox in Canada in the Watts-Strogatz network. The study period for Canada starts from May 18, 2022. The total number of nodes in Canada is 30,000,000 excluding 19% of people over 65 years old according to Statistics Canada [33]. We parameterize the model using the MCMC approach on the basis of new and cumulative confirmed cases reported by the Our World in Data and analyze the impact of heterogeneity networks on the transmission of Mpox outbreaks [32].

**Fig. 3:**
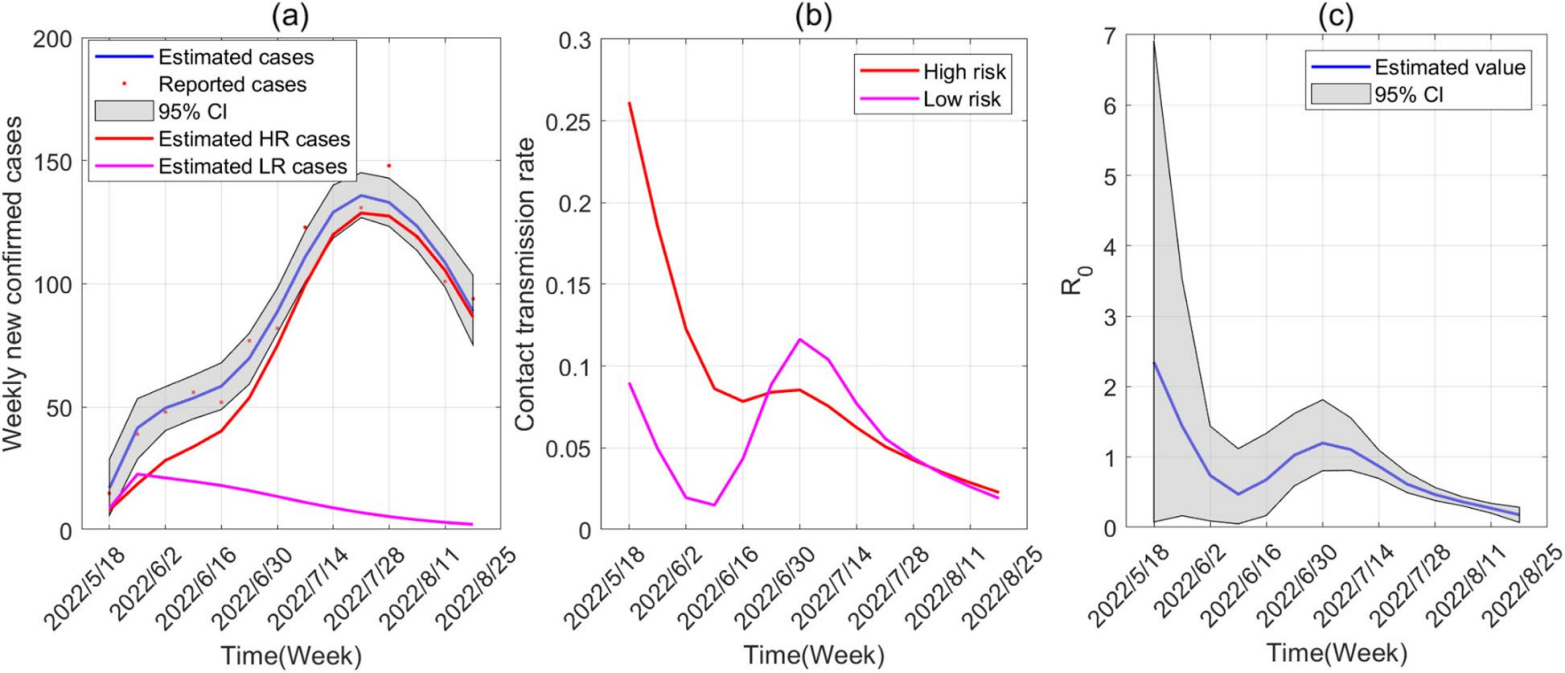
**a** Fitting the weekly new confirmed cases in the WS network from May 18, 2022, to August 18, 2022. **b** Contact transmission rates of high-risk groups and low-risk groups. **c** The basic reproduction number. In this figure and the following figure, the shaded areas represent 95% confidence intervals

We simulate the spread of Mpox in Canada on the WS network. Based on the estimated parameters in Table 2, Fig. 3a illustrates a fitting result of Model (1) and the reported Mpox cases from May 18, 2022, to August 18, 2022, in Canada. The blue solid curve shows the median value of all 50,000 simulated outputs. The shaded region is the 95% confidence interval, and the red and pink solid curves are the numbers of confirmed cases in HR and LR, respectively. Figure 3b shows the contact rates among humans. The contact rates of HR from 0.2653 to 0.0230 shows a decreasing trend, except a small oscillation in the fifth week. The contact rates of the LR reaches a peak after a rapid decline, with the highest peak reaching 0.1141 and the lowest value being 0.0149. Following Model (1), the basic reproduction number $R_{0}$ becomes (see Additional file 1)

$$R_{0} = \max \left\{ {\beta_{h} \left( {c_{hh} + c_{lh} } \right)\left( {\frac{{\xi_{1} }}{{\eta_{h} }} + \frac{1}{{\gamma_{h} + d_{h} }}} \right)\frac{{\left\langle {k^{2} } \right\rangle }}{\left\langle k \right\rangle },\frac{{\beta_{a} }}{{\gamma_{a} + d_{a} }}} \right\}.$$

**Table 2:** The parameter values and initial values are obtained by fitting weekly confirmed cases of Mpox in Canada using the MCMC

Parameter	Mean value	Std	95% CI	Reference
$\beta_{h}$(Phase 1)	0.4149	0.2716	[0.0161, 0.9184]	MCMC
$\beta_{h}$(Phase 2)	0.2333	0.1556	[0.0133, 0.6116]	MCMC
$\beta_{h}$(Phase 3)	0.2153	0.0851	[0.0925, 0.4473]	MCMC
$\beta_{h}$(Phase 4)	0.1432	0.0543	[0.0741, 0.2726]	MCMC
$\beta_{h}$(Phase 5)	0.0835	0.0639	[0.0125, 0.2343]	MCMC
$c_{hh}$(Phase 1)	0.6305	0.2490	[0.0780, 0.9834]	MCMC
$c_{hh}$(Phase 2)	0.3693	0.2997	[0.0155, 0.9750]	MCMC
$c_{hh}$(Phase 3)	0.3967	0.2396	[0.0224, 0.8966]	MCMC
$c_{hh}$(Phase 4)	0.3545	0.2727	[0.0151, 0.9478]	MCMC
$c_{hh}$(Phase 5)	0.2715	0.2331	[0.0097, 0.8877]	MCMC
$c_{lh}$(Phase 1)	0.2165	0.1238	[0.0181, 0.4706]	MCMC
$c_{lh}$(Phase 2)	0.0639	0.0518	[0.0029, 0.2074]	MCMC
$c_{lh}$(Phase 3)	0.5406	0.2394	[0.0814, 0.9274]	MCMC
$c_{lh}$(Phase 4)	0.3886	0.2546	[0.0170, 0.9473]	MCMC
$c_{lh}$(Phase 5)	0.2285	0.2082	[0.0093, 0.7471]	MCMC
$\xi_{1}$	0.3772	0.2546	[0.0180, 0.9088]	MCMC
$\beta_{ah1}$	0.3242	0.2487	[0.0221, 0.8961]	MCMC
$\beta_{ah2}$	0.2592	0.2180	[0.0106, 0.7817]	MCMC
$E^{h1} \left( 0 \right)$	21	15	[0.7149, 53.1491]	MCMC
$I^{h1} \left( 0 \right)$	4	2	[0.1445, 8.4714]	MCMC
$E^{h2} \left( 0 \right)$	51	23	[4.7388, 91.9819]	MCMC
$I^{h2} \left( 0 \right)$	4	3	[0.1767, 9.0356]	MCMC
$E^{a} \left( 0 \right)$	30	22	[1.6413, 75.9028]	MCMC
$I^{a} \left( 0 \right)$	6	4	[0.2387, 18.3195]	MCMC

Figure 3c shows the trends of the reproduction number. The reproduction number is below one from June 2 to June 16 and after July 14, while reaching a maximum of 2.3475 (95% CI: 0.0749–6.9084) on May 18, 2022.

**Fig. 4:**
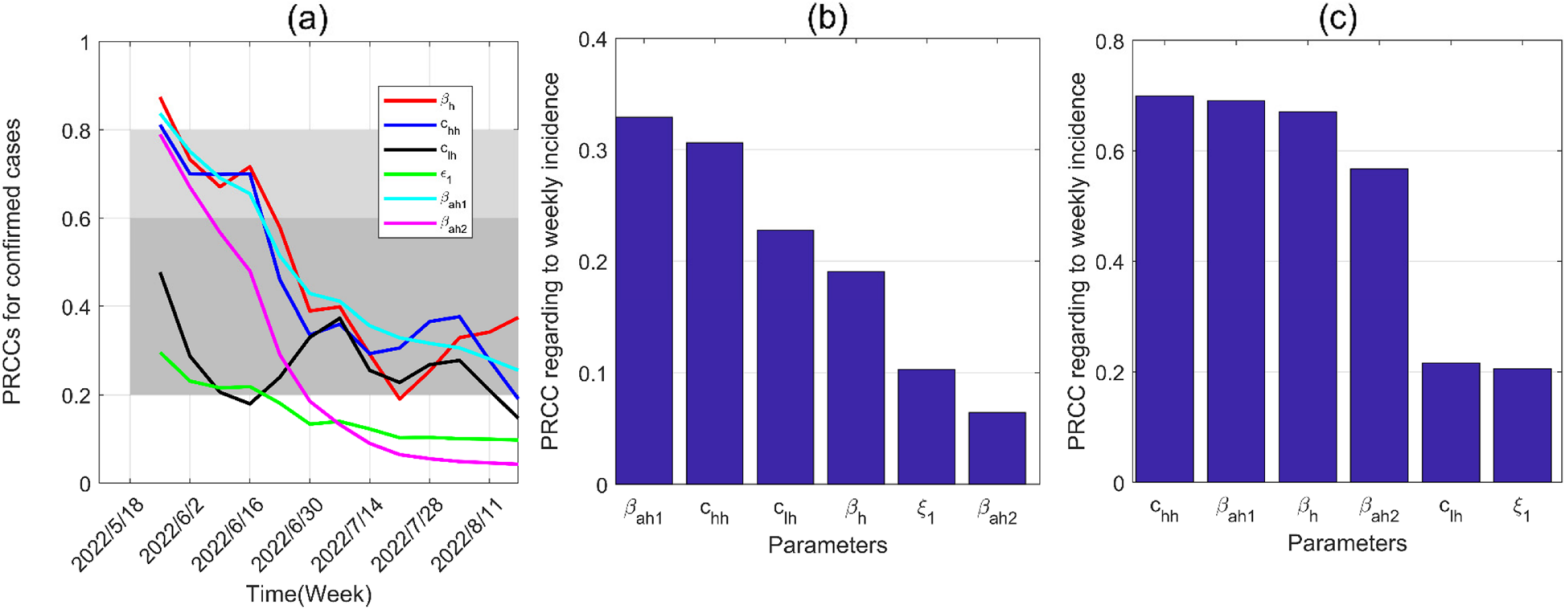
Sensitivity analysis of weekly confirmed cases with respect to transmission parameters. **a** Sensitivity analysis from May 18, 2022, to August 18, 2022. **b** Sensitivity analysis from June 1, 2022 to June 7, 2022. **c** Sensitivity analysis from July 20, 2022 to July 26, 2022

**Fig. 5:**
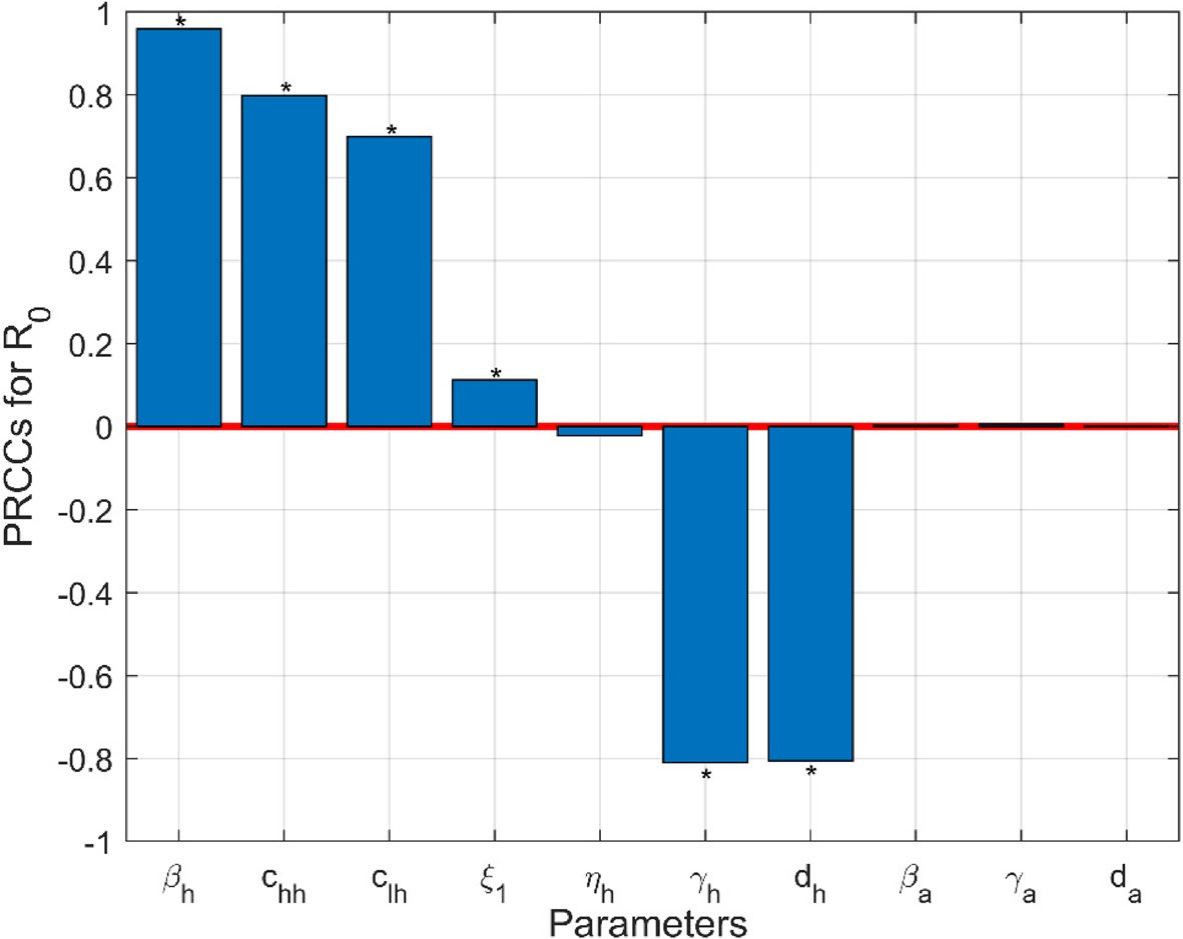
Sensitivity analysis of $R_{0}$ with respect to transmission parameters

We carry out a sensitivity analysis for all input parameters according to the weekly new confirmed cases in Fig. 4 to further identify the factors affecting the weekly new confirmed cases. As is shown, weekly number of new confirmed cases is more sensitive to $\beta_{ah1} ,\beta_{h} ,c_{hh}$, and $c_{lh}$. In contrast, the sensitivity analysis of the basic reproduction number $R_{0}$ with respect to transmission parameters in Fig. 5 reveals that the contact rate among HR $\beta_{h}$ is the predominant factor influencing, followed in significance by recovery rate of humans ($\gamma_{h}$), mortality rate of humans ($d_{h}$), probability of transmission per contact among HR ($c_{hh}$), and probability of transmission per contact from HR to LR ($c_{lh}$).

**Fig. 6:**
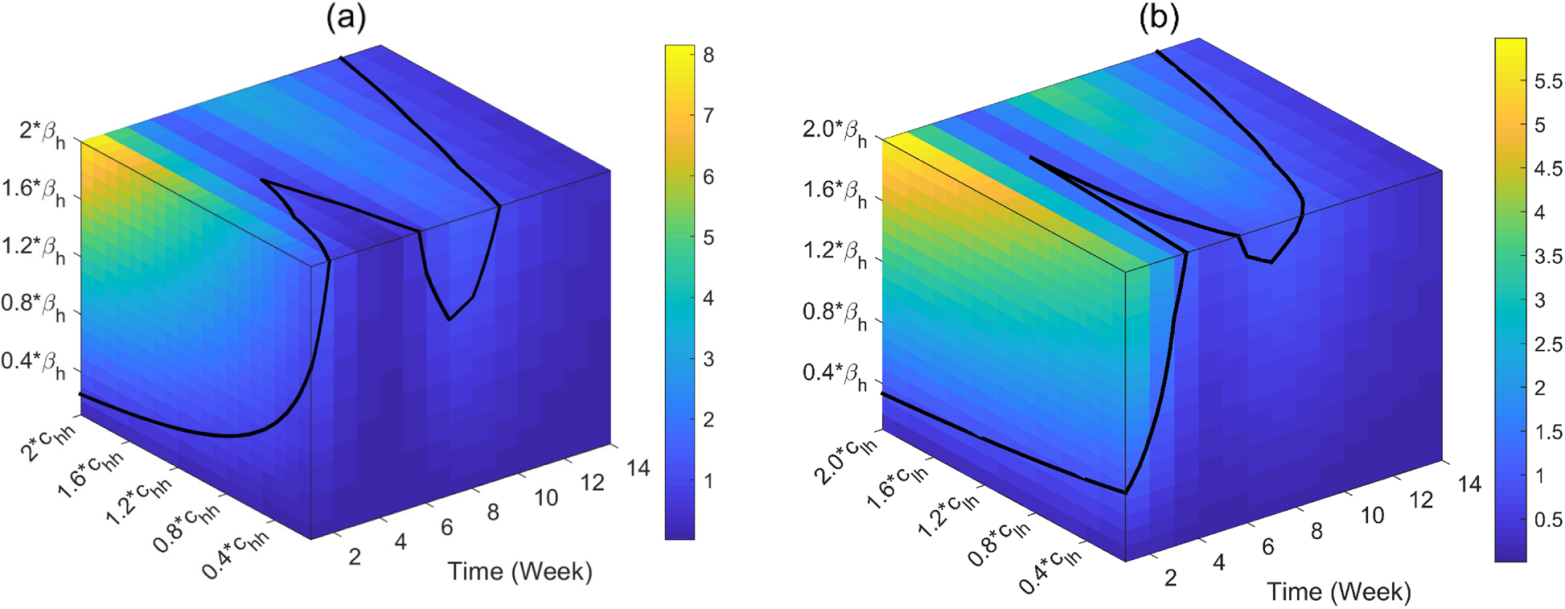
Trends of the basic reproduction number $R_{0}$ varying with in parameters $c_{hh}$, $c_{lh}$ and $\beta_{h}$. **a** Impact of parameters $c_{hh}$ and $\beta_{h}$ on the basic reproduction number $R_{0}$. **b** Impact of parameters $c_{lh}$ and $\beta_{h}$ on the basic reproduction number $R_{0}$. The right-side legend corresponds to the values of $R_{0}$, and the black solid line represents $R_{0} = 1$

Figure 6 (a) and (b) are the effect of the values of parameters $c_{hh} ,c_{lh}$, and $\beta_{h}$ on the basic reproduction number $R_{0}$, respectively. The values of the parameters are selected from the fitted values in Table 2 as the baseline values, and the range is from 0.2 to 2. Figure 6 (a) and (b) show that if the contact rate between the two groups is also relaxed, the relaxed restriction of the HR causes a greater $R_{0}$ (about 8). When $\beta_{h}$ is 0.2 times of the baseline value (mean value is 0.0436), $R_{0}$ can fall below one.

### Numerical results

We evaluate the impact of mitigation strategies on the number of cases before and after August 18, 2022, to provide a reference for implementing effective mitigation strategies in other epidemic areas. We quantify the impact of mitigation strategies for animals and humans on the number of cases through numerical simulations.

### Evaluating the mitigation strategies

**Fig. 7:**
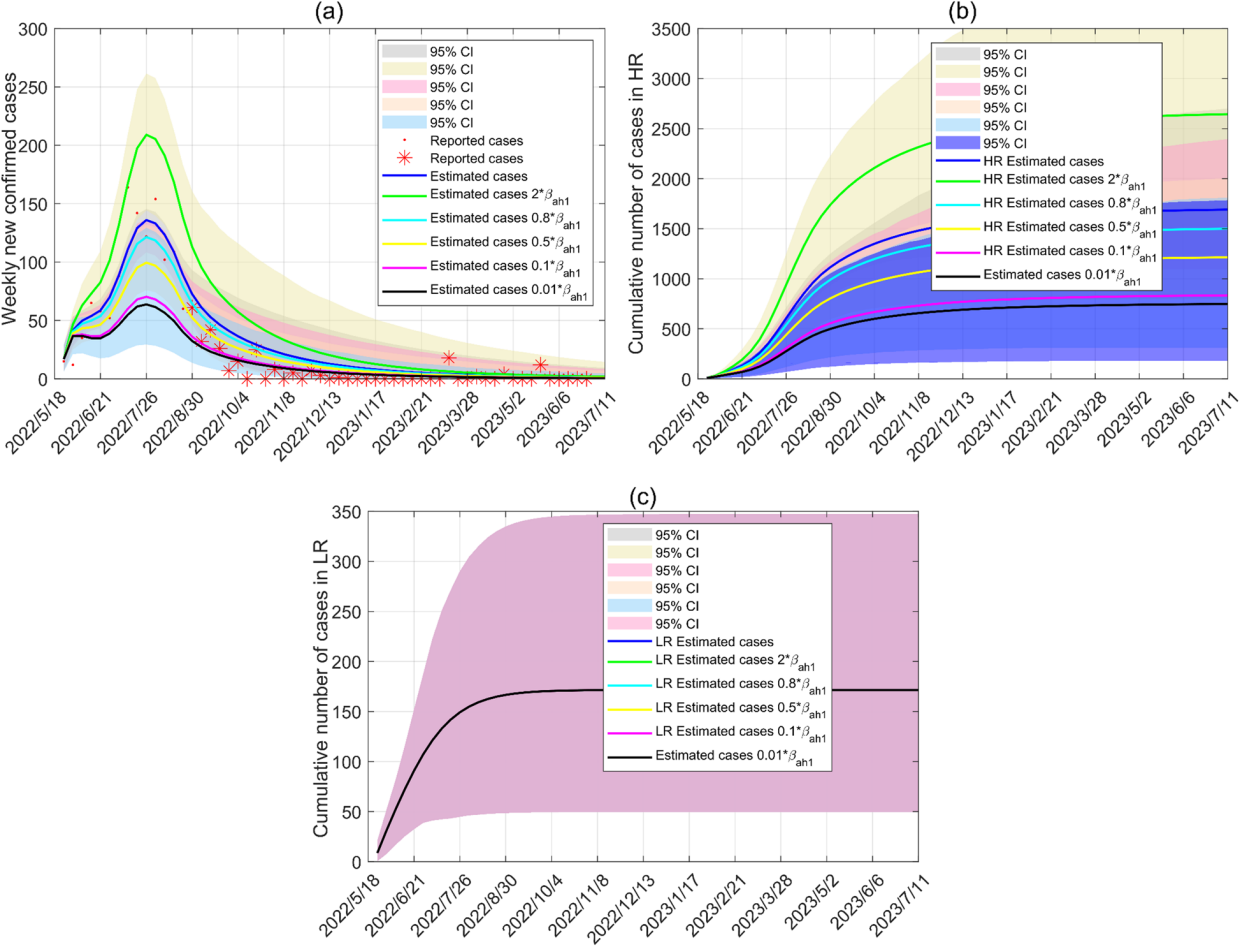
Estimated cases of animal-human contact mitigation strategies in the WS network. **a** Weekly new cases of mitigation strategies. **b** Cumulative cases in high-risk groups of mitigation strategies. **c** Cumulative cases in low-risk groups of mitigation strategies. Red dots and stars represent weekly new cases of Canada. From May 18 to August 18, 2022, the blue solid line is curve fitting, and other lines show trends in weekly new cases due to changes in transmission rates (increases of 100% and decreases of 20%, 50%, 90%, 99%) since the emergence of cases. The epidemic trend after 18 August 2022 is the result of taking mitigation strategy persistently as of 18 August 2022

**Fig. 8:**
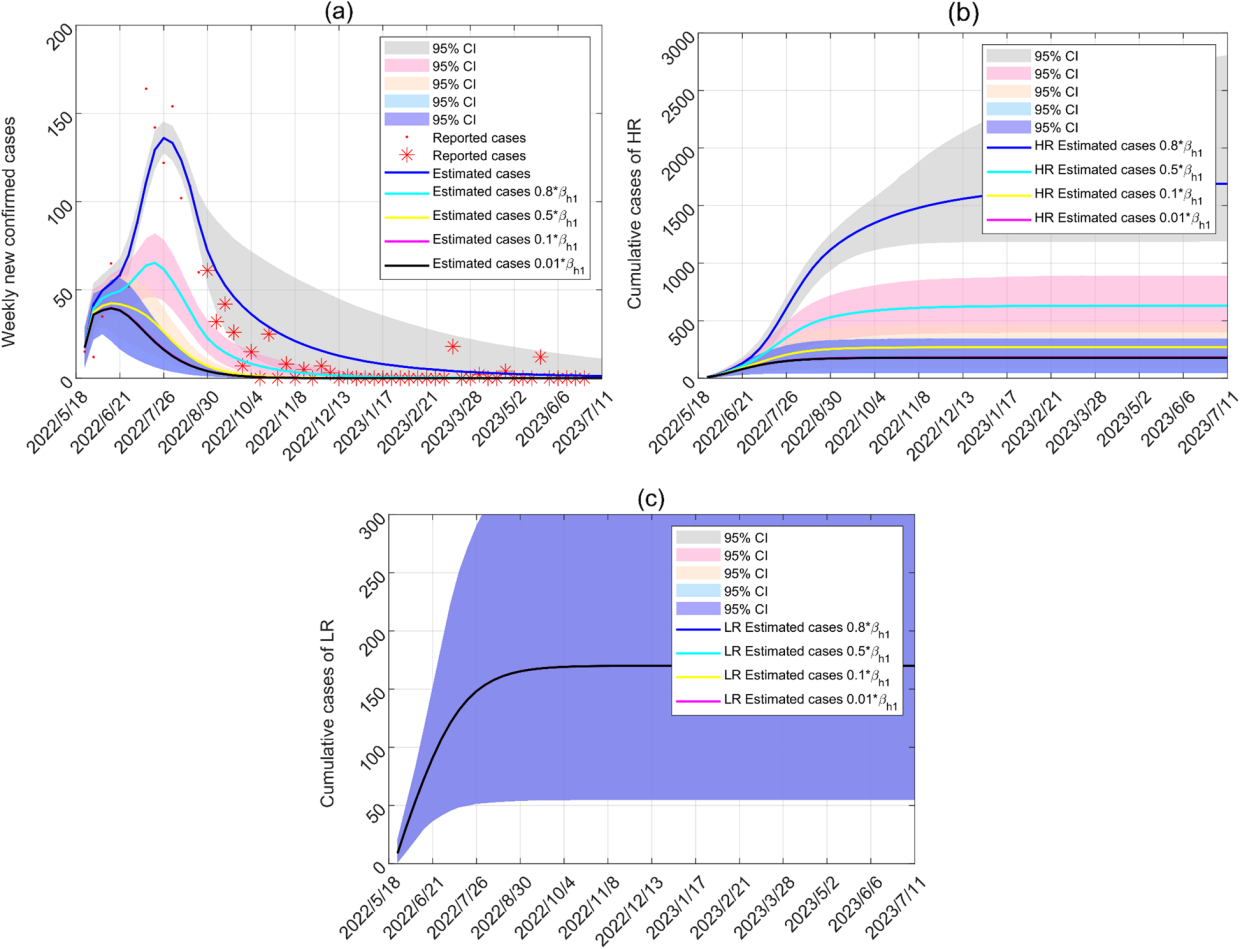
Estimated cases of mitigation strategies about sexual contacts in the WS network. **a** Weekly new cases of mitigation strategies. **b** Cumulative cases in high-risk groups of mitigation strategies. **c** Cumulative cases in low-risk groups of mitigation strategies

Our model is utilized to analyze how mitigation strategies affect the course of disease transmission. Figures 7 and 8 show the impacts of mitigation strategies with animals and HR in Canada on the WS network. By reducing the transmission from infected animals and high-risk groups by 99%, the peak of new cases has decreased by 53.54% and 70.89%, respectively. After August 18, 2022, the contact rate between infected animals and susceptible humans, depending on mitigation strategies, is reduced by approximately 20%. If the contact rates between HR and animals are reduced by 90%, the final size of the cases is constrained to 999, but the actual number of cases is 1496.

Different levels of protection for sexual contacts between infected HR and susceptible humans in Fig. 8 shorten the time to the epidemic peek of new cases. If the sexual contacts between infected HR and susceptible humans are reduced by 99%, the highest peak appearing on June 16, 2022, which is 6 weeks earlier.

**Fig. 9:**
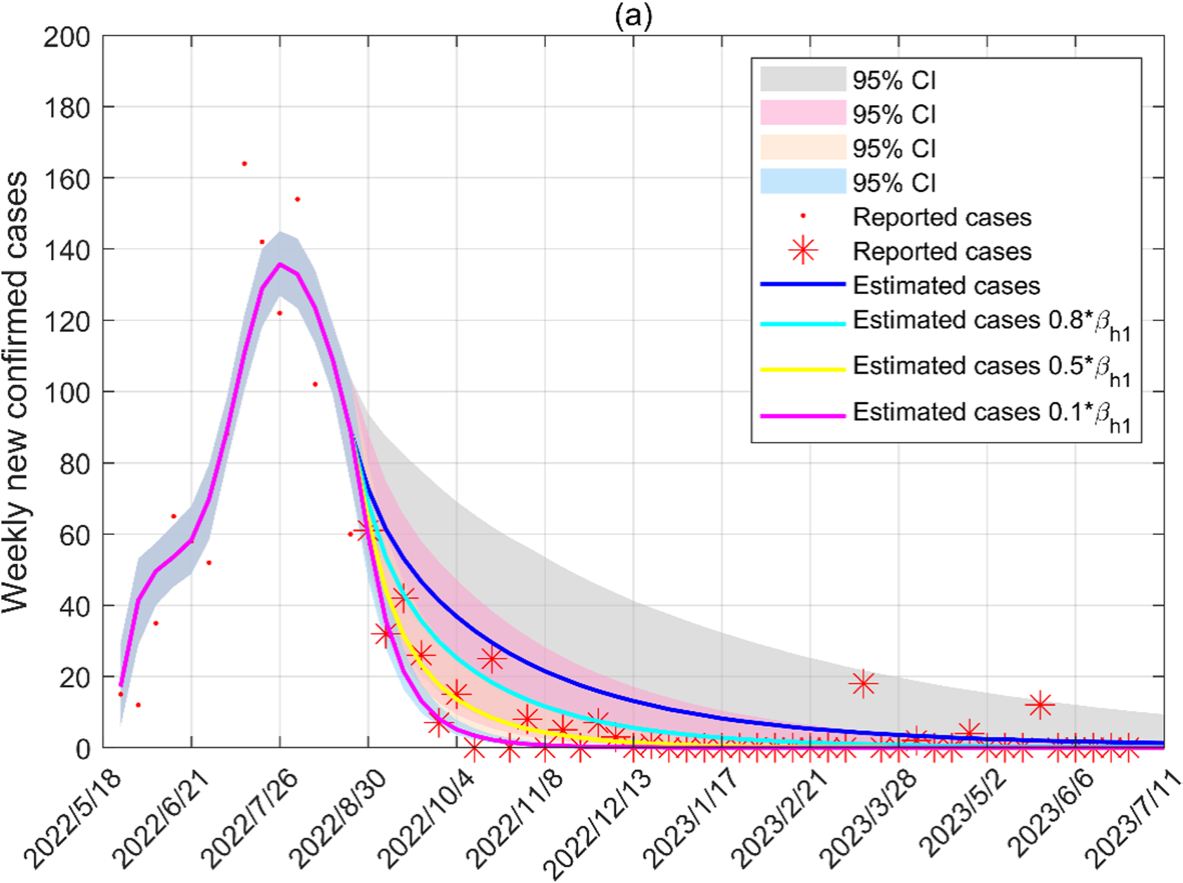
Estimated number of cases after August 18, 2022 for implementation of mitigation strategies to reduce sexual contacts (decreases of 20%, 50%, 90%)

Combined with the results of Figs. 8 and 9, the contact rates among HR reduce by 20% and 50% once a new case of Mpox appears, and the epidemic dies out 17 and 28 weeks earlier, respectively, compared with the reduction of the contact rates among HR after 18 August 2022.

### The impact of human behavior

**Fig. 10:**
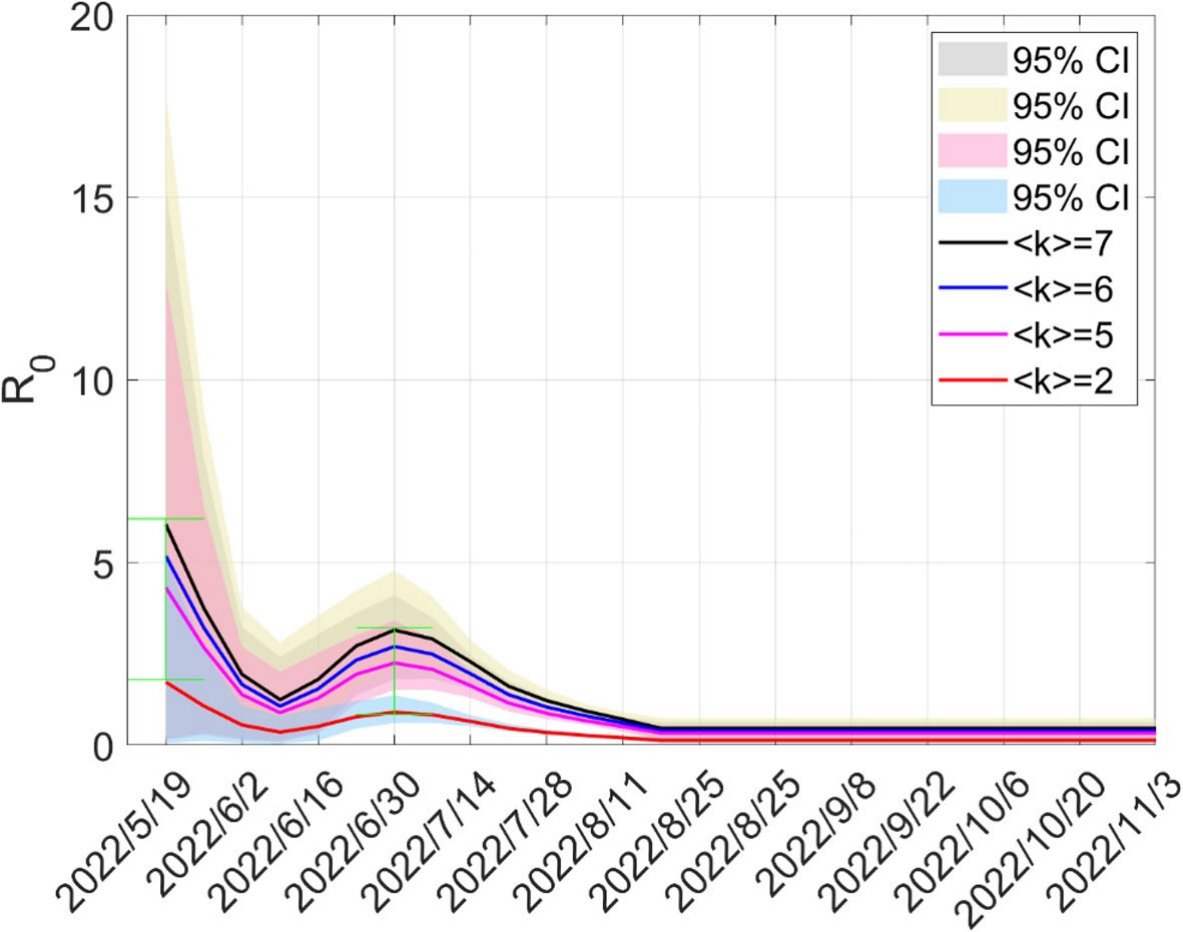
The trends of the basic reproduction number $R_{0}$ with average weekly sexual contacts $\left\langle k \right\rangle$: $\left\langle k \right\rangle = 7$ (black), $\left\langle k \right\rangle = 6$ (blue), $\left\langle k \right\rangle = 5$ (pink), $\left\langle k \right\rangle = 2$ (red)

In this section, we explore how human behavior and heterogeneity of sexual contacts affect a key epidemiological parameter—basic reproduction number $R_{0}$. Figure 10 depicts the trend of the basic reproduction number $R_{0}$ with four average node degrees. The average number of sexual contacts is reduced from seven to two every week, resulting in the basic reproduction number being below one. Therefore, increasing awareness of the risk of infection and taking protective measures in time on HR after the emergence of cases can curb the spread of MPXV.

## Discussion

Modelling the dynamic model of Mpox and evaluating mitigation strategies can optimize the allocation of resources and project mitigation strategies. We develop a network model to capture the contact heterogeneity among individuals and the role of animal hosts in transmission. The simulation takes into account the risk levels and heterogeneous contacts among humans. We analyze the potential transmission and mitigation strategies of Mpox outbreaks in Canada by using our model.

Our numerical simulations indicate that the probability of transmission per contact from HR to LR is higher than that among HR after six to 11 weeks of the appearance of new cases on May 18, 2022. Therefore, mitigation strategies should be prioritized for LR to prevent the recurrence of the epidemic and the transition of LR into HR for six to 11 weeks. The HR contact rate after May 18, 2022, is approximately 20% lower than that before May 18, 2022. If the contact rate is reduced by 90% when new cases emerge, the final epidemic size can be reduced by 76.6%. Reducing the sexual contacts among HR at the beginning of an outbreak can shorten the time to epidemic peak and lower the peak epidemic size.

The basic reproduction number is positively correlated with the level of heterogeneity among humans. The average number of sexual contacts are reduced to two per week, causing in the reproduction number to be below one. At the same time, reducing the human-to-human and animal-to-human contacts cut down the basic reproduction number and final epidemic size. Consequently, mitigation strategies should be applied for both humans and animals.

In summary, public education, control of wild animals and pets, the use of condoms, and other measures to avoid contacts with the virus can considerably reduce the scale of infection during Mpox outbreaks. Therefore, raising public awareness of these countermeasures may reduce the spread of Mpox outbreaks caused by improper personal protection. Even if confirmed cases are declining, it is necessary to continue strengthening education and media campaigns to prevent the recurrence of the epidemic caused by LR. Our model can be applied to study the transmission dynamics of Mpox in other countries or regions.

Fitting daily data into our model can project more effective mitigation strategies once they are publicly available. Animals are assumed to be homogeneous, their heterogeneity in the real world is ignored while modeling. In future work, we will provide a more comprehensive model to estimate the disease process from the following aspects. By taking into account spatial heterogeneity, we will expand the model to multi-patches to improve the accuracy of predicting the spread of infectious diseases and make more effective mitigation strategies. On the other hand, human behavior often rely on detailed information about the disease. We will develop a model of adaptive behavior in response to epidemiological perception. The epidemiological perception of the environment can adapt the behavior of susceptible humans toward mitigation strategies. Furthermore, we will determine the optimal control strategies with adaptive feedback to analyze the impact of adaptive feedback on mitigation strategies [44, 45].

## Conclusion

We propose a coupled two-layer Watts-Strogatz network to evaluate the effectiveness of mitigation strategies and control the human-to-human transmission of novel Mpox strains in the Democratic Republic of Congo in December 2023. The results indicate a positive correlation between the basic reproduction number and the level of heterogeneity in human contacts, with the basic reproduction number estimated at 2.3475 (95% CI: 0.0749–6.9084). Additionally, the probability of transmission per contact from HR to LR is higher than that among HR after six to 11 weeks of the appearance of new cases. Hence, public health agencies should urge LR to take preventive measures to avoid the recurrence of the outbreak. Reducing the average weekly frequency of sexual intercourse to two or less times will minimize the harm of Mpox on the Democratic Republic of Congo.

### Supplementary Information

**Supplementary Materials 1. **

## Data Availability

Data and materials were obtained from public domain resources as cited in the paper.

## Abbreviations

WHO: World Health Organization
MSM: Men who have sex with men
Non-MSM: Non-men who have sex with men
MPXV: Mpox virus
HR: High-risk groups
LR: Low-risk groups
MCMC: Markov chain Monte Carlo
PRCC: Partial Rank Correlation Coefficient
WS: Watts-Strogatz
