# Supplementary material for: Modelling the impact of human behavior using a two-layer Watts-Strogatz network for transmission and control of Mpox
Source: BMC Infect Dis. 2024 Mar 26;24:351. doi: 10.1186/s12879-024-09239-7 (PMC10964561; doi:10.1186/s12879-024-09239-7)
Supplement: Supplementary file 1 — Supplementary Materials 1. [file 12879_2024_9239_MOESM1_ESM.pdf]

Additional file 1

Calculation of the basic reproduction number:
System (2.2) can be rewritten as follows,

$$\begin{cases} \dot{s}_k^{h1} = -\beta_h k s_k^{h1}\Theta_{11} - \beta_{ah1}\frac{s_k^{h1}N^m}{N^h}i^a \\ \dot{e}_k^{h1} = \beta_h k s_k^{h1}\Theta_{11} + \beta_h k s_k^{h2}\Theta_{22} + \beta_{ah1}\frac{s_k^{h1}N^m}{N^h}i^a - \lambda_h e_k^{h1}, \\ \dot{p}_k^{h1} = \lambda_h e_k^{h1} - \eta_h p_k^{h1}, \\ \dot{i}_k^{h1} = \eta_h p_k^{h1} - (\gamma_h + d_h)\, i_k^{h1}, \\ \dot{r}_k^{h1} = \gamma_h i_k^{h1}, \\ \dot{s}_k^{h2} = -\beta_h k s_k^{h2}\Theta_{22} - \beta_{ah2}\frac{s_k^{h2}N^m}{N^h}i^a, \\ \dot{e}_k^{h2} = \beta_{ah2}\frac{s_k^{h2}N^m}{N^h}i^a - \lambda_h e_k^{h2}, \\ \dot{p}_k^{h2} = \lambda_h e_k^{h2} - \eta_h p_k^{h2}, \\ \dot{i}_k^{h2} = \eta_h p_k^{h2} - (\gamma_h + d_h)\, i_k^{h2}, \\ \dot{r}_k^{h2} = \gamma_h i_k^{h2}, \\ \dot{s}^a = -\beta_a s^a i^a \\ \dot{e}^a = \beta_a s^a i^a - \lambda_a e^a, \\ \dot{i}^a = \lambda_a e^a - (\gamma_a + d_a)\, i^a, \\ \dot{r}^a = \gamma_a i^a. \end{cases}$$

Here,

$$\Theta_{11} = \frac{c_{hh}\sum\limits_{k=1}^{n} kp(k)\left(\xi_1 p_k^{h1} + i_k^{h1}\right)}{\langle k\rangle}, \Theta_{22} = \frac{c_{lh}\sum\limits_{k=1}^{n} kp(k)\left(\xi_1 p_k^{h1} + i_k^{h1}\right)}{\langle k\rangle}.$$

The above system is an $10n + 4$ dimension system, where $s_k^{h1}(t) = S_k^{h1}(t)/N_k^{h1}$ means the relative density of susceptible individuals with degree k at time t in high-risk groups, $e_k^{h1}(t), p_k^{h1}(t), i_k^{h1}(t), r_k^{h1}(t), s_k^{h2}(t), e_k^{h2}(t)$, $p_k^{h2}(t), i_k^{h2}(t), r_k^{h2}(t), s^a(t), e^a(t), i^a(t), r^a(t)$ are similar to $s_k^{h1}(t)$. We quantify the total number of infected individuals by calculating epidemic thresholds to determine whether an epidemic will spread or die out. To estimate the transmission potential of the epidemic, we derive R_0 of the model, important epidemic threshold, defined as the average secondary cases produced by an infected individual in a completely susceptible

population [1]. There exists a unique disease-free equilibrium,

$$(s_1^{h1},\dots,s_n^{h1},E_1^{h1},\dots,e_n^{h1},p_1^{h1},\dots,p_n^{h1},i_1^{h1},\dots,i_n^{h1},r_1^{h1},\dots,r_n^{h1},s_1^{h2},\dots,$$
$$s_n^{h2},e_1^{h2},\dots,e_n^{h2},p_1^{h2},\dots,p_n^{h2},i_1^{h2},\dots,i_n^{h2},r_1^{h2},\dots,r_n^{h2},s^a,e^a,i^a,r^a)$$
$$=(1,\dots,1,0,\dots,0,0,\dots,0,0,\dots,0,0,\dots,0,1,\dots,1,$$
$$0,\dots,0,0,\dots,0,0,\dots,0,0,\dots,0,1,0,0,0)=:P_0.$$

We compute R_0 using the next generation matrix approach [2, 3]. The Jacobian matrix F is

$$F=\begin{bmatrix}
0_{n\times n} & \frac{\beta_h\xi_1(c_{hh}+c_{lh})}{\langle k\rangle}f_1 & \frac{\beta_h(c_{hh}+c_{lh})}{\langle k\rangle}f_1 & 0_{n\times n} & 0_{n\times n} & 0_{n\times n} & 0_{n\times 1} & \beta_{ah1}\frac{N^m}{N^h}I_1\\
0_{n\times n} & 0_{n\times n} & 0_{n\times n} & 0_{n\times n} & 0_{n\times n} & 0_{n\times n} & 0_{n\times 1} & 0_{n\times 1}\\
0_{n\times n} & 0_{n\times n} & 0_{n\times n} & 0_{n\times n} & 0_{n\times n} & 0_{n\times n} & 0_{n\times 1} & 0_{n\times 1}\\
0_{n\times n} & 0_{n\times n} & 0_{n\times n} & 0_{n\times n} & 0_{n\times n} & 0_{n\times n} & 0_{n\times 1} & \beta_{ah2}\frac{N^m}{N^h}I_1\\
0_{n\times n} & 0_{n\times n} & 0_{n\times n} & 0_{n\times n} & 0_{n\times n} & 0_{n\times n} & 0_{n\times 1} & 0_{n\times 1}\\
0_{n\times n} & 0_{n\times n} & 0_{n\times n} & 0_{n\times n} & 0_{n\times n} & 0_{n\times n} & 0_{n\times 1} & 0_{n\times 1}\\
0_{1\times n} & 0_{1\times n} & 0_{1\times n} & 0_{1\times n} & 0_{1\times n} & 0_{1\times n} & 0 & \beta_a\\
0_{1\times n} & 0_{1\times n} & 0_{1\times n} & 0_{1\times n} & 0_{1\times n} & 0_{1\times n} & 0 & 0
\end{bmatrix},$$

where I_1 is a unit vector of dimension $n*1$, and

$$f_1=\begin{pmatrix} p(1) & 2p(2) & \cdots & np(n)\\ 2p(1) & 4p(2) & \cdots & 2np(n)\\ \vdots & \vdots & \ddots & \vdots\\ np(1) & 2np(2) & \cdots & n^2p(n)\end{pmatrix}.$$

The matrices V is

$$V=\begin{bmatrix}
\lambda_hI_n & 0_{n\times n} & 0_{n\times n} & 0_{n\times n} & 0_{n\times n} & 0_{n\times n} & 0_{n\times 1} & 0_{n\times 1}\\
-\lambda_hI_n & \eta_hI_n & 0_{n\times n} & 0_{n\times n} & 0_{n\times n} & 0_{n\times n} & 0_{n\times 1} & 0_{n\times 1}\\
0_{n\times n} & -\eta_hI_n & (\gamma_h+d_h)I_n & 0_{n\times n} & 0_{n\times n} & 0_{n\times n} & 0_{n\times 1} & 0_{n\times 1}\\
0_{n\times n} & 0_{n\times n} & 0_{n\times n} & \lambda_hI_n & 0_{n\times n} & 0_{n\times n} & 0_{n\times 1} & 0_{n\times 1}\\
0_{n\times n} & 0_{n\times n} & 0_{n\times n} & -\lambda_hI_n & \eta_hI_n & 0_{n\times n} & 0_{n\times 1} & 0_{n\times 1}\\
0_{n\times n} & 0_{n\times n} & 0_{n\times n} & 0_{n\times n} & -\eta_hI_n & (\gamma_h+d_h)I_n & 0_{n\times 1} & 0_{n\times 1}\\
0_{1\times n} & 0_{1\times n} & 0_{1\times n} & 0_{1\times n} & 0_{1\times n} & 0_{1\times n} & \lambda_a & 0\\
0_{1\times n} & 0_{1\times n} & 0_{1\times n} & 0_{1\times n} & 0_{1\times n} & 0_{1\times n} & -\lambda_a & (\gamma_a+d_a)
\end{bmatrix}.$$

Therefore,

$$FV^{-1}=\begin{bmatrix}
A & A & B & 0_{n\times n} & 0_{n\times n} & 0_{n\times n} & C & C\\
0_{2n\times n} & 0_{2n\times n} & 0_{2n\times n} & 0_{2n\times n} & 0_{2n\times n} & 0_{2n\times n} & 0_{2n\times 1} & 0_{2n\times 1}\\
0_{n\times n} & 0_{n\times n} & 0_{n\times n} & 0_{n\times n} & 0_{n\times n} & 0_{n\times n} & D & D\\
0_{2n\times n} & 0_{2n\times n} & 0_{2n\times n} & 0_{2n\times n} & 0_{2n\times n} & 0_{2n\times n} & 0_{2n\times 1} & 0_{2n\times 1}\\
0_{1\times n} & 0_{1\times n} & 0_{1\times n} & 0_{1\times n} & 0_{1\times n} & 0_{1\times n} & E & E\\
0_{1\times n} & 0_{1\times n} & 0_{1\times n} & 0_{1\times n} & 0_{1\times n} & 0_{1\times n} & 0 & 0
\end{bmatrix},$$

where

$$A=\frac{\beta_h(c_{hh}+c_{lh})}{\langle k\rangle}\left(\frac{\xi_1}{\eta_h}+\frac{1}{\gamma_h+d_h}\right)f_1, B=\frac{\beta_h(c_{hh}+c_{lh})}{(\gamma_h+d_h)\langle k\rangle}f_1, C=\beta_{ah1}\frac{N^m}{N^h(\gamma_a+d_a)}I_1,$$

$$D=\beta_{ah2}\frac{N^m}{N^h(\gamma_a+d_a)}I_1, E=\frac{\beta_a}{\gamma_a+d_a}.$$

The characteristic equation of FV^{-1} is

$$\begin{aligned}&\left|\lambda I_{(8n+10)\times(8n+10)}-FV^{-1}\right|\\ =&\begin{vmatrix}\lambda I_{n\times n}-A & -A & -B & 0_{n\times n} & 0_{n\times n} & 0_{n\times n} & -C & -C\\ 0_{n\times n} & M & 0_{n\times n} & 0_{n\times n} & 0_{n\times n} & 0_{n\times n} & 0_{n\times n} & 0_{n\times n}\\ 0_{n\times n} & 0_{n\times n} & M & 0_{n\times n} & 0_{n\times n} & 0_{n\times n} & 0_{n\times n} & 0_{n\times n}\\ 0_{n\times n} & 0_{n\times n} & 0_{n\times n} & M & 0_{n\times n} & 0_{n\times n} & -D & -D\\ 0_{n\times n} & 0_{n\times n} & 0_{n\times n} & 0_{n\times n} & M & 0_{n\times n} & 0_{n\times n} & 0_{n\times n}\\ 0_{n\times n} & 0_{n\times n} & 0_{n\times n} & 0_{n\times n} & 0_{n\times n} & M & 0_{n\times n} & 0_{n\times n}\\ 0_{n\times n} & 0_{n\times n} & 0_{n\times n} & 0_{n\times n} & 0_{n\times n} & 0_{n\times n} & \lambda-E & -E\\ 0_{n\times n} & 0_{n\times n} & 0_{n\times n} & 0_{n\times n} & 0_{n\times n} & 0_{n\times n} & 0 & \lambda\end{vmatrix}=0,\end{aligned}$$

where

$$M=\begin{pmatrix}\lambda & \cdots & 0\\ \vdots & \ddots & \vdots\\ 0 & \cdots & \lambda\end{pmatrix}_{(n+1)\times(n+1)}.$$

The characteristic equation of FV^{-1} is equal to

$$\lambda^{5n+1}(\lambda-E)\left|\lambda I_{n\times n}-A\right|=0,$$

Therefore, the similarity matrix has the same eigenvalue, we have

$$f_1=\begin{pmatrix}p(1) & 2p(2) & \cdots & np(n)\\ 2p(1) & 4p(2) & \cdots & 2np(n)\\ \vdots & \vdots & \ddots & \vdots\\ np(1) & 2np(2) & \cdots & n^2p(n)\end{pmatrix}_{n\times n}\sim\begin{pmatrix}f_a & 2p(2) & \cdots & np(n)\\ 0 & 0 & \cdots & 0\\ \vdots & \vdots & \ddots & \vdots\\ 0 & 0 & \cdots & 0\end{pmatrix}_{n\times n},$$

where

$$f_a=p(1)+2^2p(2)+\cdots+n^2p(n)=\left\langle k^2\right\rangle.$$

The basic reproduction number R_0 becomes

$$R_0=\max\left\{\beta_h\left(c_{hh}+c_{lh}\right)\left(\frac{\xi_1}{\eta_h}+\frac{1}{\gamma_h+d_h}\right)\frac{\left\langle k^2\right\rangle}{\langle k\rangle},\frac{\beta_a}{\gamma_a+d_a}\right\}.$$

References

[1] Grey JA, Bernstein KT, Sullivan PS, Purcell DW, Chesson HW, Gift TL, et.al. Estimating the population sizes of men who have sex with men in US states and

counties using data from the American Community Survey. JMIR Public Hlth Sur. 2016;2(1):e5365.

[2] Van den Driessche P, Watmough J. Further notes on the basic reproduction number. Math Epidemiology. 2008;1945:159–178.

[3] Diekmann O, Heesterbeek JAP, Metz JA. On the definition and the computation of the basic reproduction ratio R_0 in models for infectious diseases in heterogeneous populations. J Math Biol. 1990;28:365–382.
